# Inducing a mental context for associative memory formation with real-time fMRI neurofeedback

**DOI:** 10.1038/s41598-022-25799-7

**Published:** 2022-12-08

**Authors:** Silvy H. P. Collin, Philip L. C. van den Broek, Tim van Mourik, Peter Desain, Christian F. Doeller

**Affiliations:** 1 Tilburg School of Humanities and Digital Sciences, Tilburg University, Tilburg, The Netherlands; 2 Donders Institute for Brain, Cognition and Behaviour, Radboud University, Nijmegen, The Netherlands; 3 Max Planck Institute for Human Cognitive and Brain Sciences, Leipzig, Germany; 4 Kavli Institute for Systems Neuroscience, Centre for Neural Computation, The Egil and Pauline Braathen and Fred Kavli Centre for Cortical Microcircuits, Jebsen Centre for Alzheimer’s Disease, Norwegian University of Science and Technology, Trondheim, Norway; 5 Institute of Psychology-Wilhelm Wundt, Leipzig University, Leipzig, Germany

**Keywords:** Cognitive neuroscience, Learning and memory

## Abstract

Memory, one of the hallmarks of human cognition, can be modified when humans voluntarily modulate neural population activity using neurofeedback. However, it is currently unknown whether neurofeedback can influence the integration of memories, and whether memory is facilitated or impaired after such neural perturbation. In this study, participants memorized objects while we provided them with abstract neurofeedback based on their brain activity patterns in the ventral visual stream. This neurofeedback created an implicit face or house context in the brain while memorizing the objects. The results revealed that participants created associations between each memorized object and its implicit context solely due to the neurofeedback manipulation. Our findings shed light onto how memory formation can be influenced by synthetic memory tags with neurofeedback and advance our understanding of mnemonic processing.

## Introduction

Humans can be trained to voluntarily modulate neural activity in various brain regions, which has been shown to influence behavior^1–13^. Animal studies using optogenetics have demonstrated that modulating neurons can influence memory^14,15^. Furthermore, human intracranial studies have shown that behavior is influenced when participants voluntarily modulate activity of single neurons in the medial temporal lobe (MTL)^16^, or the functional Magnetic Resonance Imaging (fMRI) signal using neurofeedback^17–20^. For example, by using real-time fMRI neurofeedback to associate basic visual features in early visual cortex^18,19^ or to improve sustained attention ability^17^. However, it remains unknown if neurofeedback can influence the integration of memories of more complex visual stimuli.

Memory has been shown to depend on the context in which to-be-remembered material was studied^21,22^. Training participants to voluntarily modulate their neural activity in a particular way (e.g., by providing neurofeedback based on what information was represented in the brain) would lead to better control over the mental context of the participants during neural modulation^7,9,13,17,19,23^. Additionally, memory performance has been shown to be vulnerable to interference from other (related) memories^24–26^. Suthana et al. showed that intracranial neural modulation in the entorhinal region during encoding enhanced memory, in contrast, Jacobs et al. showed the opposite effect using intracranial neural modulation as well. Possibly, using a task that is known to rely heavily on the entorhinal cortex combined with the specific timing of the neural modulation might have induced interfering memories coming to mind which then caused memory impairment of to-be-remembered information in Jacobs et al.^5^. Due to contrasting findings like Jacobs et al.^5^ and Suthana et al.^3^, it remains unclear under what circumstances neurofeedback would facilitate memory.

**Figure 1:**
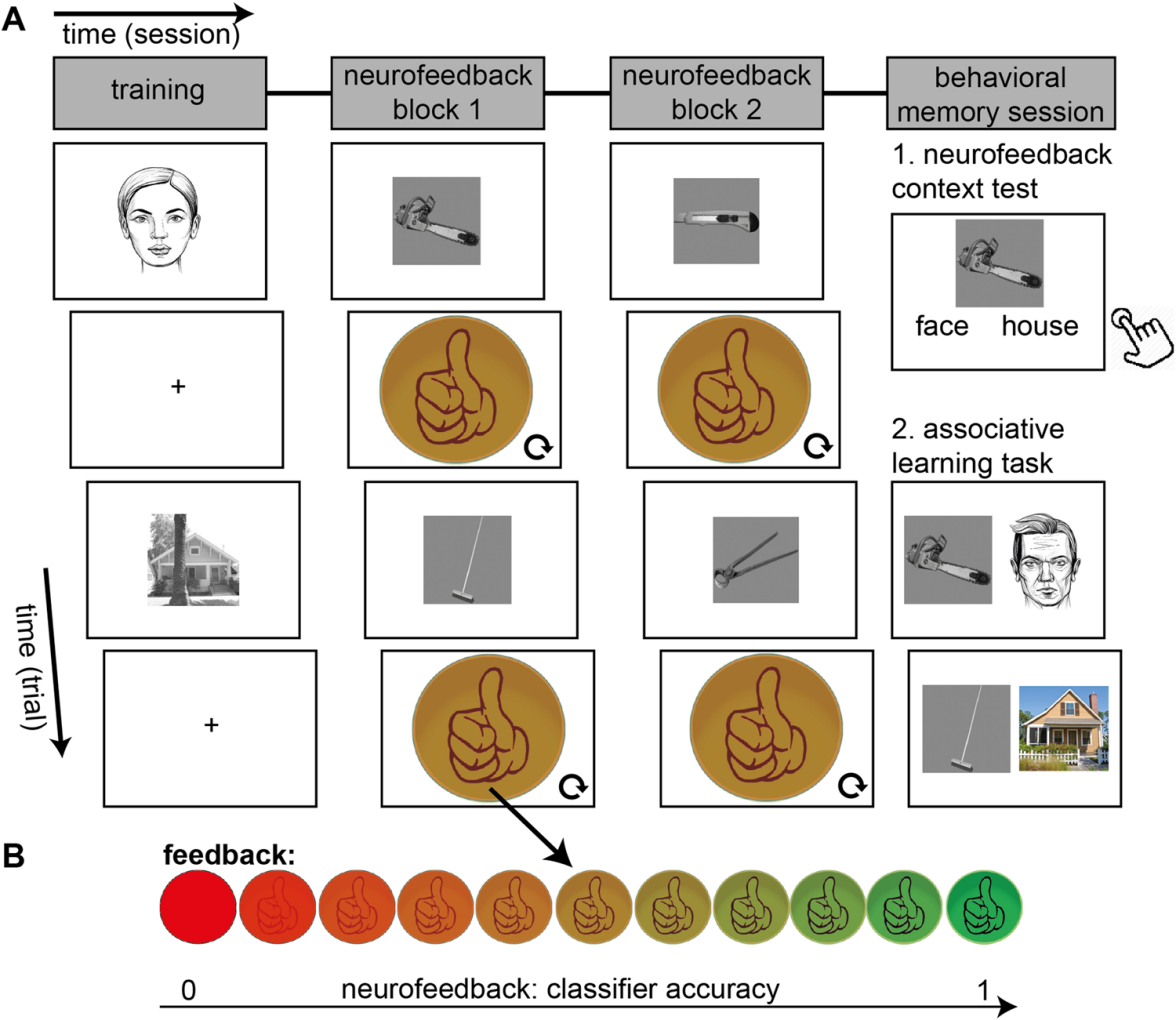
Structure of experimental sessions and neurofeedback. (**A**) Overview of the experiment. The experiment started with an MRI-session that included a training block, and two neurofeedback blocks. The training block contained pictures of faces and of houses, and was used to train a classifier on brain activity patterns evoked by faces and houses. During the two neurofeedback blocks, each trial (24 s duration) started with an image of an object (for 2 s), and was then followed by an abstract presentation of the face (one block) or house (the other block) classifier accuracy [see panel (**B**)]. The MRI-session was followed by a behavioral memory session that included a neurofeedback context test in which memory was tested for the artificial context created by the neurofeedback. The neurofeedback context test was a two-alternative forced choice task during which the participants were presented with the same objects as during the neurofeedback blocks, and had to indicate for each object whether it belongs to faces or to houses (with a button press). Subsequently participants were presented with an associative learning task. During the associative learning task, each of the 32 objects from the neurofeedback blocks was associated with either a specific face or a specific house. Half of the objects were associated with a specific exemplar (face/house) of the same category as they received neurofeedback on in the MRI-session, and the other half of the objects were associated with a specific exemplar from the other category as they received neurofeedback on. After learning of these pairs, they filled in a Vividness of Visual Imagery Questionnaire, followed by a memory test. Here, it was tested for each object whether they remembered which category it was associated with, and subsequently, with which specific exemplar it was associated. (**B**) Neurofeedback. For each trial, the neurofeedback started with an orange circle in which a hand with a thumb up was presented. Based on the classifier accuracy, the alpha-level of the image was adapted, and could therefore change into a red circle (low classifier accuracy) or a green circle with a clearly visible thumb (high classifier accuracy). In one block, the classifier accuracy corresponded to the face category, and in the other block to the house category. The order of the two blocks were counterbalanced across participants.

In this experiment, we investigated whether providing feedback based on neural activity in the ventral visual stream can facilitate associative memory using non-invasive fMRI in humans. The experiment (see Fig. 1) started with an fMRI-session that included a training block, and two neurofeedback blocks. The training block contained pictures of faces and houses, and was used to train a classifier to distinguish brain activity patterns evoked by those stimulus categories. During the two neurofeedback blocks, each trial started with an image of an object, and was then followed by an abstract presentation of the face (one block) or house (the other block) classifier evidence (see Fig. 1B). The order of these two neurofeedback blocks was unknown to the participants. This manipulation trained participants to voluntarily modulate their neural activity, and created an implicit ‘face mental context’ in one block, and ‘house mental context’ in the other block. The MRI-session was followed by a behavioral memory session that included a neurofeedback context test in which memory was tested for the artificial context created by the neurofeedback, and subsequently an associative learning task. The neurofeedback context test was a two-alternative forced choice task during which the participants were presented with the same objects as during the neurofeedback blocks, and had to indicate for each object whether it belongs to faces or to houses. During the associative learning task, each of the objects from the neurofeedback blocks was associated with a specific exemplar (face or house). Half of the objects were associated with a specific exemplar (face/house) of the same category as they received neurofeedback on in the fMRI-session, and the other half of the objects were associated with a specific exemplar from the other category as they received neurofeedback on. We tested their memory of these associations for the category (face or house) and the specific exemplar.

We hypothesized that it is possible to create a category-specific mental context in higher order visual regions by neurofeedback. We predicted that this mental context would be accompanied by significant decoding evidence of the associated category after participants were exposed to the neurofeedback for a sufficient time, i.e., at the end of each of the neurofeedback blocks. We predicted that the (during neurofeedback) studied objects will become associated with the specific context (face or house) in which they are being encoded^22,27^. Furthermore, we predict that subsequent use of the same objects in an associative memory task will facilitate associative memory for the category, and furthermore modulate memory for the specific associations, either by facilitating associative learning^3^ or by interfering with associative learning^5^.

## Results

### Real-time decoding from higher order visual regions

**Figure 2:**
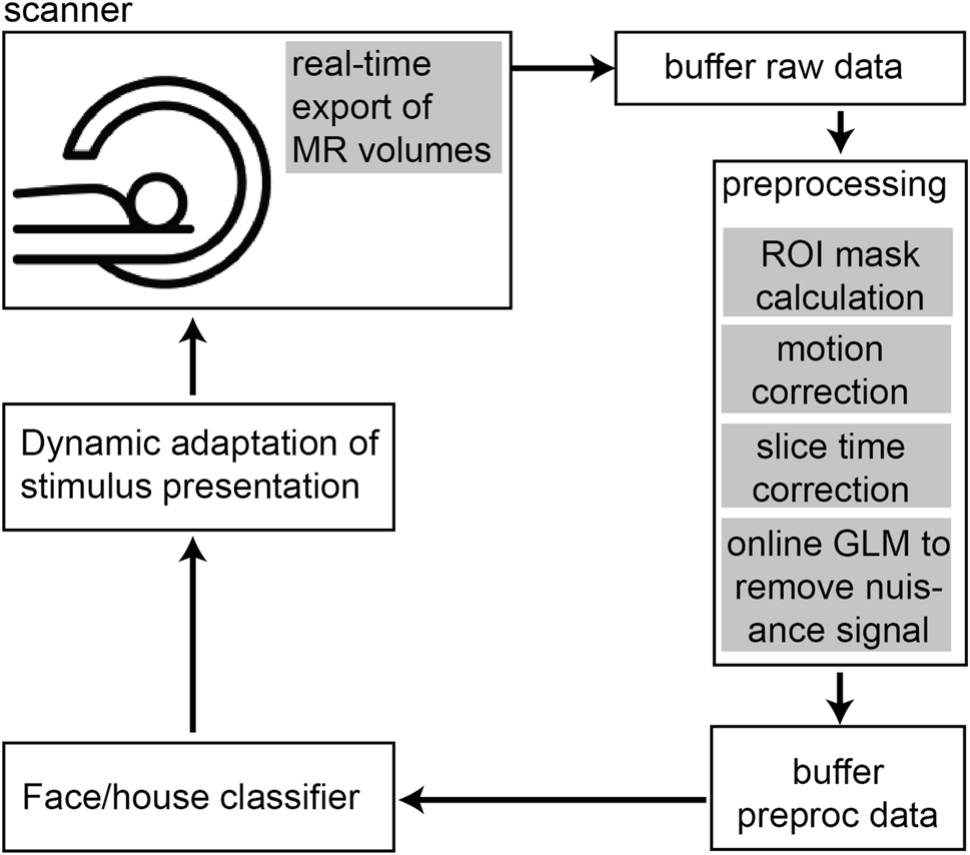
Technical set-up. The technical set-up used for real-time fMRI as used by Ref.^28^. The data was exported in real-time into a FieldTrip buffer, and then immediately preprocessed. A structurally defined ROI mask was calculated. The mask and preprocessed data were imported into BrainStream for online decoding analysis. See “Methods” for more details.

**Figure 3:**
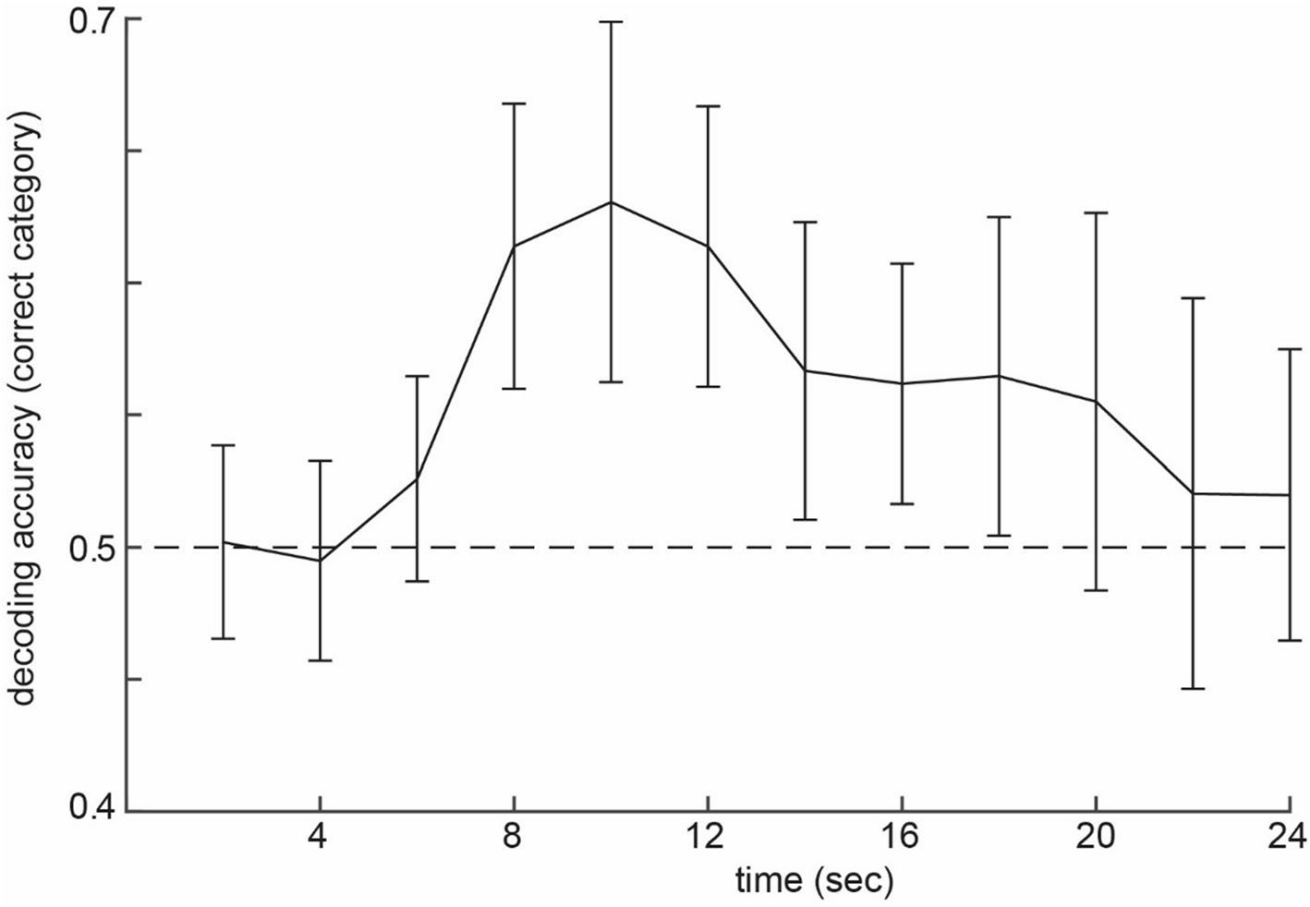
Group average of the decoding evidence during the test phase of the pilot experiment. Decoding evidence (group average ± SEM) of the correct category (0.5 is chance level, indicated by the dashed line). The object is presented on the screen the first 2 s.

Before conducting a study using classifier evidence from higher order visual regions (i.e. parahippocampal gyrus and fusiform gyrus, see Methods) as neurofeedback, it is important to establish a technical set-up (see Fig. 2) that would reliably classify an associated item (when it is not presented on the screen) at a single subject, volume-by-volume level. This was verified in a separate pilot experiment (see Supplementary Information, and Supplementary Figs. 1 and 2). Based on these pilot results, we predicted to find increased classifier evidence for the correct category in the neurofeedback trials of the main experiment from Time-in-trial (i.e., MR volume) 4 to 9 (see Fig. 3), which is why we focused our MR analyses on the neurofeedback trials in the main experiment on these time points.

### Neurofeedback

**Figure 4:**
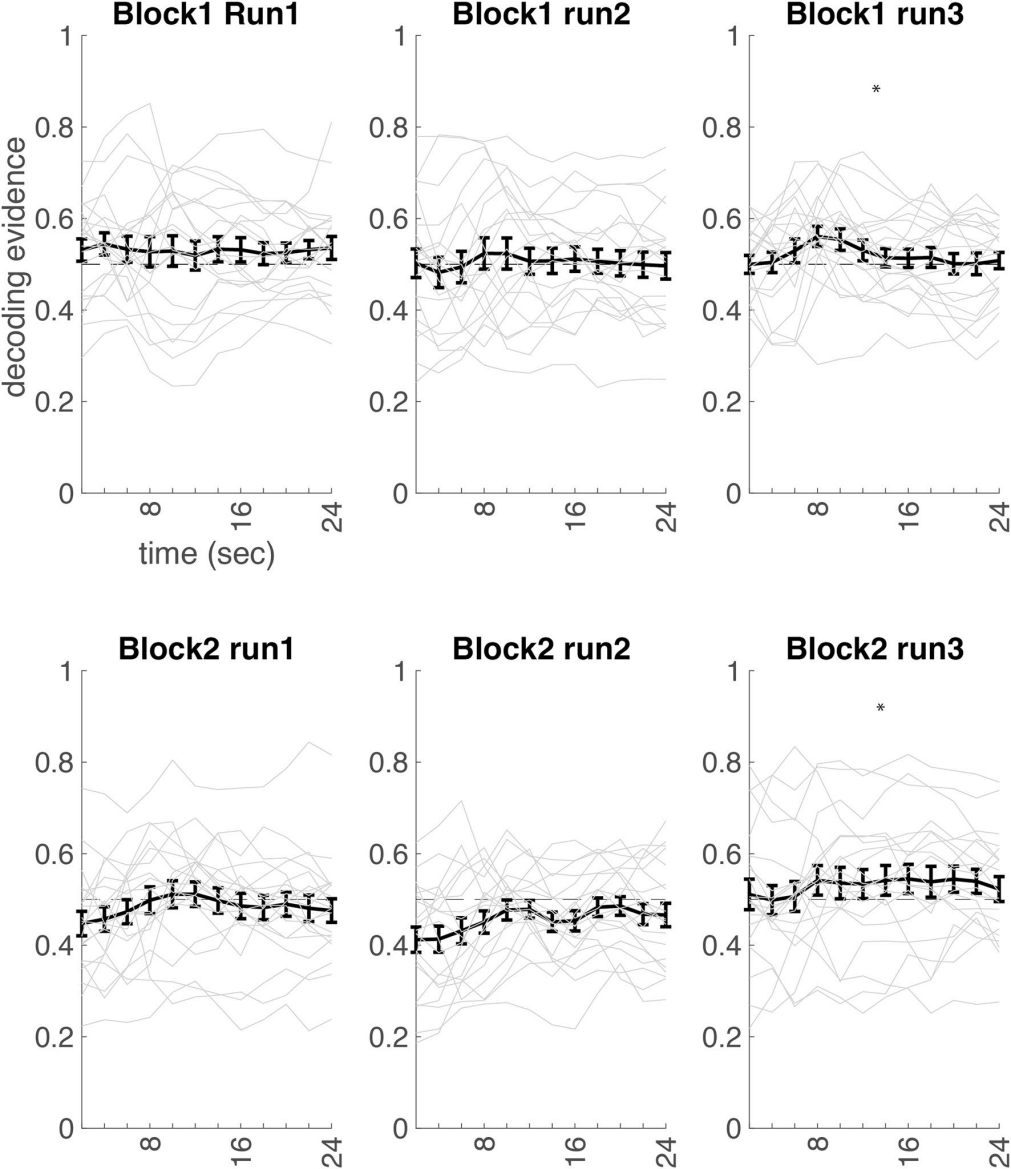
Decoding evidence. Decoding evidence of the correct category (face or scene) during neurofeedback over time, thus, separately for block 1 and 2, and separately for run 1, 2 and 3 (averaged across all trials of that run). Blocks are labeled block 1 and 2 since it is counterbalanced across participants which of the two blocks is face neurofeedback and which is scene neurofeedback. 0.5 is chance level, indicated by the dashed line. Error bars represent SEM. Grey lines represent single-subject data. The object is presented on the screen the first 2 s, the neurofeedback presentation (see Fig. 1B) is presented in the remaining 22 s.

A repeated measures anova with run (1, 2, 3), block (1, 2) and time (time-points 4–9, as selected based on pilot study, see results section “Real-time decoding from higher order visual regions”) as within-subject factors showed a run × block × time interaction effect F (10,190) = 2.585, p = 0.006. Therefore, separate repeated measures anova’s with block (1,2) and time (4–9) as within-subject factors were performed for each of the three runs. Run 3 showed a significant main effect (i.e., above chance classification accuracy across the run) F (1,19) = 4.490, P = 0.047. Run 1 and 2 did not show such a main effect (run1: F (1,19) = 0.432, p = 0.519; run2: F(1,19) = 0.375, p = 0.548). Thus, as expected, above chance classifier evidence for the correct category (i.e., faces in one block and houses in the other block, based on what was being trained on with the neurofeedback) was present at the end of each neurofeedback block, in run 3 (see Fig. 4). Note that this effect was present even though the participants were not told when they have to imagine faces and when they had to imagine scenes.

### Neurofeedback facilitated associative memory

**Figure 5:**
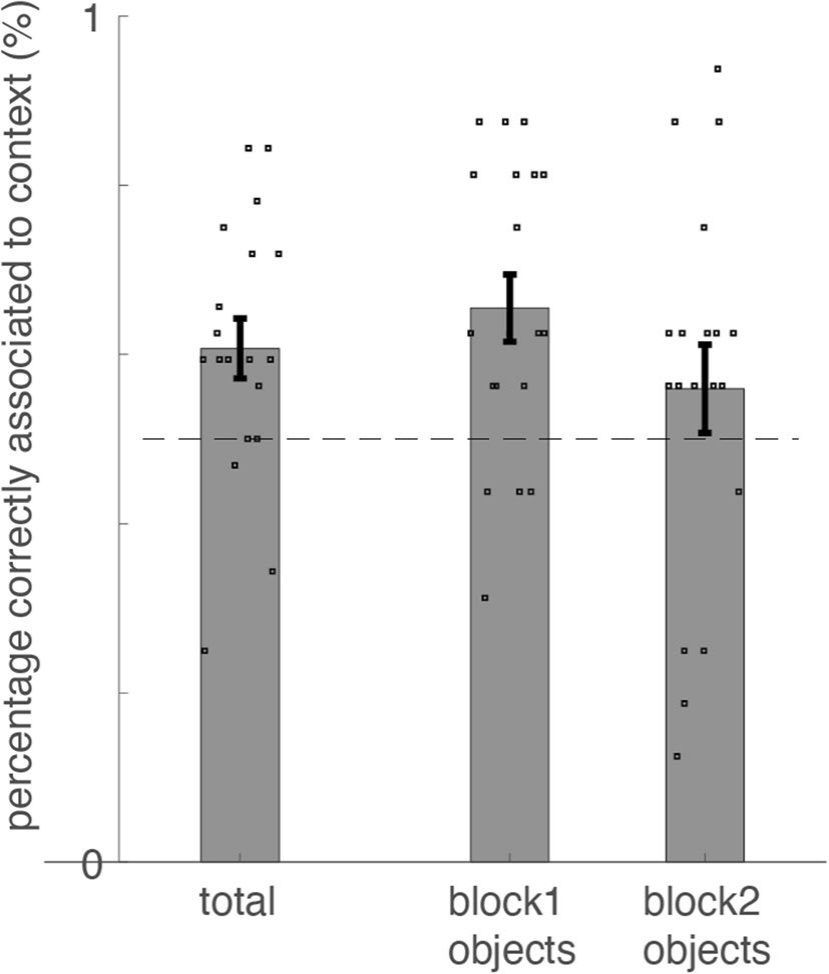
Percentage of correct responses during the neurofeedback context test. Average (± SEM) percentage of correct responses during the neurofeedback context test (overlaid with individual responses). Blocks are labeled block 1 and 2 since it is counterbalanced across participants which of the two blocks is face neurofeedback and which is scene neurofeedback. From left to right: average across all objects, average across those objects studied during neurofeedback block 1, average across those objects studied during neurofeedback block 2. One subject was an outlier and therefore excluded (i.e. more than 2.5 SD from group mean). Total: T (1,18) = 3.007, P = 0.008.

Strikingly, the neurofeedback context test revealed that participants indeed associated the memorized objects with their corresponding neurofeedback context. Thus, half of the memorized objects became associated with the face-context induced by neurofeedback, and the other half of the memorized objects became associated with the house-context induced by neurofeedback. It was counterbalanced across participants which object became associated by neurofeedback with which category (T (1,18) = 3.007, P = 0.008, see Fig. 5).

**Figure 6:**
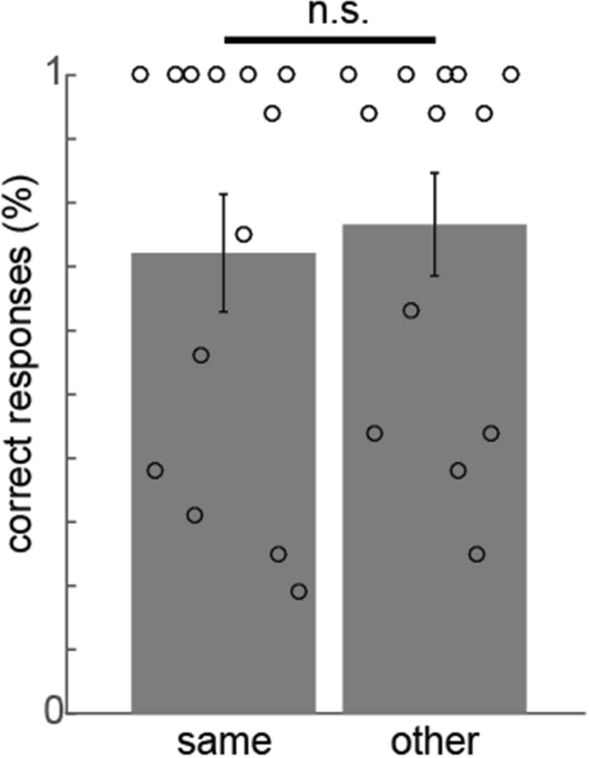
Results of the subsequent associative learning task. Average (± SEM) percentage of correct responses during the subsequent associative learning task, plotted separately for same category pairs and other category pairs (overlaid with individual responses). There was no significant difference between same and other category pairs’ memory.

During the subsequent associative learning task (see Fig. 6), participants had to memorize pairs of images, consisting of the same objects as used in the neurofeedback blocks but now explicitly associated with a novel face or house. Each object was either associated with the same or the other category compared to the neurofeedback context. We predicted that it would modulate associative memory when an object would be associated with the same context as the initial neurofeedback context. However, there was no difference between same category pairs and other category pairs for category memory (T (1,19) = 0.448, p = 0.659) nor for exemplar memory (T (1,19) = − 1.406, p = 0.176).

## Discussion

In this study, participants memorized objects while we provided them with neurofeedback to have their brain activity patterns in ventral visual stream regions represent either faces or houses simultaneously to memorizing the objects. The results revealed that the neurofeedback manipulation led to the creation of a category specific mental context in the ventral visual stream. Crucially, this caused participants to associate the memorized objects with its specific mental context, either face of house, solely based on abstract neurofeedback.

The fMRI neurofeedback manipulation led to modulation of across-voxel neural representations in ventral visual stream regions in such a way that it would represent faces in one half of the experiment and houses in the other half of the experiment, creating an implicit face and house context while having to memorize objects. These face and house representations emerged solely by the continuously adapted, abstract presentation of individual face-house classifier output as feedback to the participants. Thus, crucially, without explicitly notifying them when to think about what category. This is in line with findings from earlier studies in which it has been demonstrated that participants can learn to voluntarily control their across-voxel neural patterns by neurofeedback^7,17–19,28^. Importantly, our results reveal the possibility of solely using neurofeedback to train participants to associate (more complex) stimulus categories in higher order regions to objects, which extends the neurofeedback study from Amano et al.^19^ in which an association between a color and a grating orientation was created in the early visual cortex by training participants to modulate across-voxel neural patterns in their early visual cortex to represent a specific color while presenting a specific orientation on the screen.

Additionally, we tested how the neurofeedback influenced associative learning. The results revealed that the mental context solely created by neurofeedback caused associative learning, i.e. objects became associated to their corresponding context as shown by a post-scanning memory test. Memories are contextualized, and remembering something will lead to recovery of the context in which that memory was formed^29^. Empirical research showing the importance of context for memory^30–32^ dates back to the famous Godden and Baddeley study showing the influence of a land vs underwater context on memorizing words^33^. Less is known about how malleable the neural underpinnings of a memory context is^30,34^. These results demonstrate the malleability of a mental context by showing that it is possible to modulate the neural representations of a mental context to represent a specific image category solely by providing participants with abstract feedback based on their neural activity patterns while another task is being performed (in our case learning a list of objects). Furthermore, we provide evidence for the notion that this mental (face/house) context implicitly influences (object) memories that are being formed in this context.

A second goal of this study was to discover whether neurofeedback would facilitate memory during a subsequent associative learning task using novel images, based on intracranial studies in humans that revealed that memory performance after modulating MTL neurons could lead to both memory facilitation^3^ as well as memory impairment^5^, possibly depending on the nature of the task performed as well as the timing of neural modulation. However, in contrast to what we expected based on earlier literature, we did not observe an effect of the neurofeedback manipulation on a subsequent associative learning task with novel images. It could possibly be due to participants approaching ceiling level performance in this task that we did not confirm our prediction. Future research would be necessary to draw more firm conclusions about this outstanding question.

We used a basic memory integration task for this experiment, as a proof-of-principle regarding the ability to use fMRI neurofeedback for memory integration. It would be interesting to do future research with more naturalistic study designs and stimuli to be able to draw conclusions regarding more complex real-world situations. Another interesting avenue of future research would be to examine what specific brain region can best be used for neurofeedback during a memory integration task, rather than, as we did here, use a large proportion of the ventral visual stream. To resolve the debate regarding when memory is facilitated and when it is impaired due to neural modulation, it would be important to use more complex associative memory tasks than the task we used, either by using more complex stimuli and study designs or by increasing the time between the neurofeedback manipulation and the subsequent memory task. For example, a naturalistic task in which it is tested whether fMRI neurofeedback can be used to ‘tag’ stimuli to be part of a specific type, much like how files are tagged on a computer. Also, the individual differences regarding people’s ability to use neurofeedback to create a context in mind are interesting to explore in future research, which is not possible to do with our current study due to the rather limited number of participants tested for such an individualized purpose.

In short, our study investigated how paired associate learning is influenced by an implicit artificial context that was created by voluntary modulation of neural populations. The results open interesting avenues for future research investigating how to influence memory integration and inference with neurofeedback, and enhances our understanding of mnemonic coding in the brain.

## Methods

### Participants

Thirty students from the Radboud University campus in Nijmegen participated in this study. All participants were right-handed and had normal or corrected-to-normal vision. Ten participants had to be excluded to do technical problems, excessive head motion, or an incomplete dataset. Thus, the final group of participants contained twenty students (nine males, aged 20–44 years, mean age 27.2). All participants gave written informed consent. Participants were informed during the informed consent procedure that their data as acquired during the experiment could be used for publication or on conferences in an anonymized form. The study was approved by the local ethics committee (CMO, Arnhem/Nijmegen). All experiments were performed in accordance with the guidelines and regulations set by the local ethics committee (CMO, Arnhem/Nijmegen, protocol 2014/288).

### Task design

The experiment consisted of a training block, two neurofeedback blocks of 3 runs each, a neurofeedback context test, and an associative learning task (see Fig. 1). The tasks were presented using Brainstream/Psychtoolbox (training block and neurofeedback blocks) or Presentation software (neurofeedback context test and associative learning task; Neurobehavioral Systems, version 16.4).

### Training block

The training block was used to train a classifier on brain activity patterns evoked by faces and houses. The training block consisted of 28 mini-blocks in total, interleaved mini-blocks with images of faces and mini-blocks with images of scenes. Each mini-block lasted for 30 s and was followed by a fixation cross which was presented for 12 s. Each mini-block consisted of 14 unique pictures (i.e., each mini-block had a different set of pictures), each picture was presented for 2 s, and, additionally, the first picture of the mini-block was repeated at a random position within that mini-block. Thus, in total participants viewed 392 unique stimuli in the training block (28 mini-blocks × 14 unique pictures per mini-block). Participants had to press a button when they saw the first picture being repeated. They were asked to maintain attending the images throughout the entire block.

### Neurofeedback blocks

Participants received two neurofeedback blocks. In block one they were presented with object stimulus 1–16 and during block two they received object stimulus 17–32. Each block consisted of three runs. During each run, each object stimulus of that neurofeedback block was shown once. The two neurofeedback blocks, participants received neurofeedback based on the evidence of the classifier after being presented with an image of an object. The object was presented during the first 2 s, and the neurofeedback was being presented for the subsequent 22 s. Each neurofeedback block had 16 unique objects that were all presented three times (all 16 objects are shown once in a random order before the first object is being repeated), which led to a total of 32 unique objects. The neurofeedback was presented in an abstract fashion; by manipulating the color of a circle, and the visibility of a hand with the thumb pointing up (see Fig. 1B). If the decoding evidence of the correct category was above chance level (i.e. above 50%), then the alpha level of the green neurofeedback image (i.e. the image with the green circle that includes an image of a hand with the thumb pointing up) increased with 0.05. If the decoding evidence of the correct category was below chance level, the alpha level of the green neurofeedback image decreased with 0.05 (making the circle appear more red, and the hand less visible). For one block the correct category was ‘face’, and for the other block the correct category was ‘house’, with the order counterbalanced across participants. This leads to 16 objects only being presented during neurofeedback training of the face category, and the other 16 objects being presented only during neurofeedback training of the house category.

### Neurofeedback instruction

Participants were instructed that the neurofeedback image will change based on their brain activity, and that they can influence this by thinking about either faces or houses. They were not told which actual category they have to think about in which block, and were told to figure this out based on the neurofeedback that they receive. Furthermore, they were told to try to vividly imagine images of that category during the neurofeedback blocks, and that they receive 3 euros extra monetary compensation with good performance throughout the entire task. The expected 5–6 s delay in the neurofeedback (due to the BOLD response) was explained to them as well. They are also told to remember which objects are shown.

### Neurofeedback context test

After the real-time fMRI session, participants were placed behind a computer screen, and conducted a neurofeedback context test. During this (self-paced) test, participants were presented with the 32 objects from the neurofeedback blocks, one at the time. For each object, they were asked to which category this object belonged (face or house).

### Associative learning task

Afterwards participants performed an associative learning task. They were presented with 32 pairs. Each pair consisted of one object and one face, or one object and one house. The faces and houses used were different from the ones used in the training block. The objects were the same as the ones used in the neurofeedback blocks. Half of the objects were associated with an exemplar (face or house) from the same category as the category on which they received neurofeedback for this object (referred to as same category pairs). The other half of the objects were associated with an exemplar (face or house) from the other category as the category on which they received neurofeedback for this object (referred to as other category pairs). There were twelve repetitions of each pair (in random order) leading to 384 trials in total, with each trial showing the images for 2.8 s and a fixation cross for 0.2 s. After a short break during which participants filled in a Vividness of Visual Imagery Questionnaire, they were tested for their memory for the 32 pairs by asking them for each of the 32 objects whether it the object belongs to a face or to a house, and subsequently to which specific face or house this object belongs. Responses were given with button presses. For the second question they received 16 possible answer options (i.e. the 16 exemplars, 8 faces and 8 houses, that were associated with one of the objects from neurofeedback block 1 were the answer options for all neurofeedback block 1 objects, and similar for neurofeedback block 2 answer options). They select their answer with a button press. The objects were presented in a random order.

### Image acquisition

All images were acquired using a 3 T Siemens Prisma scanner equipped with a 32-channel head coil (Siemens, Erlangen, Germany). The structural T1-weighted image was acquired using an MPRAGE-grappa sequence with the following parameters: TR = 2300 ms; TE = 3.03 ms; flip angle = 8°; in-plane resolution = 256 × 256 mm; number of slices = 192; acceleration factor PE = 2; voxel resolution = 1 mm^3^, duration = 321 s. The functional images were acquired using a 2D Echo Planar Imaging (EPI) sequence, with the following parameters: voxel size 3.3 × 3.3 × 3 mm, TR = 2000 ms, TE = 30 ms, flip angle = 80 deg, Multi-Band acceleration factor = 2, FOV = 212 × 212 × 105 mm.

### Real-time fMRI analysis

The functional volumes were preprocessed and analyzed in real-time using BrainStream software (see www.brainstream.nu) as was used before, see Ref.^28^, which is a Matlab-based software package developed at the Donders Centre for Cognition (Nijmegen, Netherlands). The toolbox builds on Psychtoolbox combined with an extension (StimBox) for adaptive stimulus presentation, FieldTrip toolboxes for raw and preprocessed data buffers, FSL and SPM8 for MR data analyses, a GUI streamer to access and export the raw MR volumes during acquisition, and the Donders Machine Learning Toolbox for online decoding. See Fig. 3 for an overview of the technical set-up.

### Online image preprocessing

Each functional volume was sent to another computer directly after acquisition of the volume, and stored in a Fieldtrip buffer. From this buffer, the scan entered a (matlab based) preprocessing pipeline (BrainStream). This preprocessing pipeline included motion correction (X, Y, Z, pitch, roll, yaw), slice time correction, and an online GLM to remove nuisance signal.

### Online decoding

After preprocessing, the scans entered another Fieldtrip buffer from which they entered the decoding analysis. For the training block the scans were first shifted for 6 s to account for the hemodynamic delay. Then, the scans were labeled according to their category (face, scene) and used to train a classifier. We used logistic regression in conjunction with an elastic net regularizer, as used in Niazi et al.^28^. Using a coordinate gradient-descent algorithm^35^, classifier training took only a few minutes to complete (see Ref.^28^ for more details on the classifier).

### ROI

**Figure 7:**
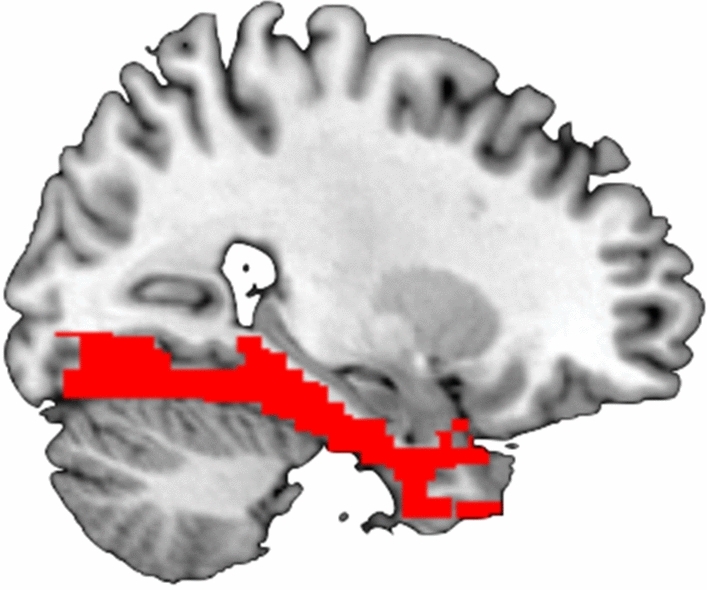
Brain image with the ROI. ROI used in this experiment was a mask of parahippocampal gyrus and fusiform gyrus, as shown in red in this brain image.

The voxels corresponding to the parahippocampal gyrus and fusiform gyrus (Fig. 7) were used for classifier training and test of both neurofeedback blocks. The WFU pickatlas was used to select voxels corresponding to parahippocampal gyrus/fusiform gyrus in MNI space. A native space mask was then calculated for each participant individually using the inverse normalization parameters to inverse an MNI space mask including parahippocampal gyrus and fusiform gyrus into subject specific space.

## Supplementary Information

Supplementary Information.

## Data Availability

The raw datasets generated and analyzed during the current study are not publicly available due to the nature of the informed consent that was acquired from participants, but are available from the corresponding author on reasonable request.
