# Supplementary material for: Inducing a mental context for associative memory formation with real-time fMRI neurofeedback
Source: Sci Rep. 2022 Dec 8;12:21226. doi: 10.1038/s41598-022-25799-7 (PMC9731952; doi:10.1038/s41598-022-25799-7)
Supplement: Supplementary file 1 — Supplementary Information. [file 41598_2022_25799_MOESM1_ESM.pdf]

Supplementary information

Supplementary pilot experiment

Before conducting a study using classifier evidence as neurofeedback to train participants to integrate memories, it is important to establish a technical set-up that would reliably classify an associated item at a single subject, single trial, volume-by-volume level from our regions of interest, which is the aim of this pilot experiment. We used a basic associative memory design, and adapted that to a real-time fMRI decoding version, in order to test whether we could reliably decode stimulus categories at a single subject, single trial, volume-by-volume level at moments that *not* the being decoded stimulus category but an *associated* item was presented on the screen (see Supplementary Fig. 1 for task design).

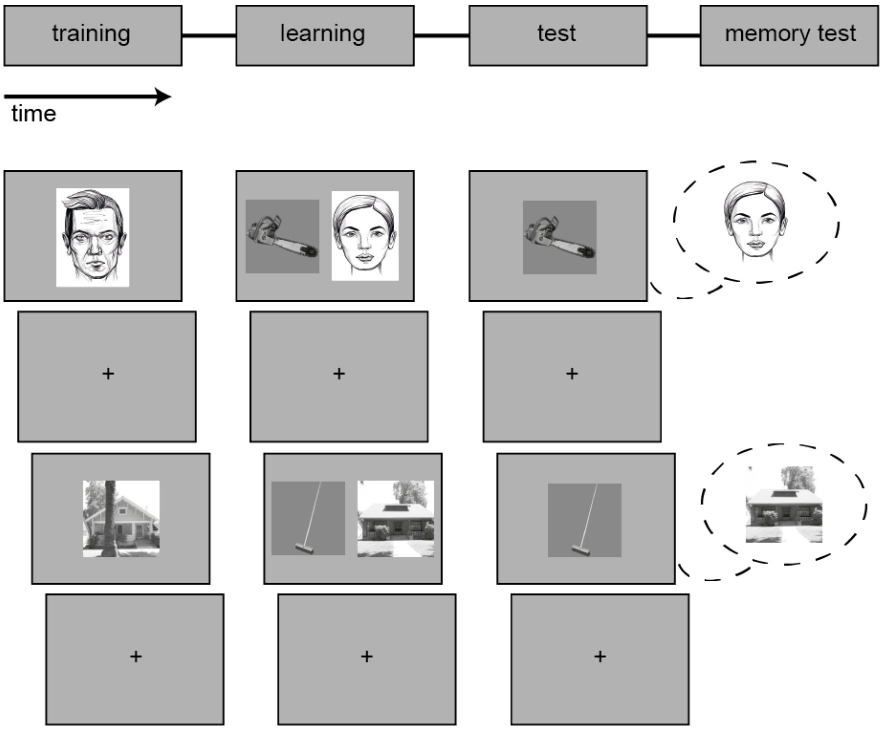

Supplementary Figure 1: Experimental design – pilot experiment

The pilot experiment consisted of a training phase, learning phase, test phase and memory test.

Results

We trained a classifier on brain activity patterns evoked by faces and houses. In a learning phase, participants learned four object-face pairs, and four object-house pairs. Subsequently, participants were presented with these eight objects (consecutively in a random order) while we calculated for each MR volume the decoding accuracy of faces and houses from their brain activity patterns (see methods for more details on task and analyses).

The classifier results for scene and face decoding showed variable results across trials/participants. Of the 20 trials per category (5 participants, 4 trials each), scene decoding was above chance level (i.e. 0.5) on 17 out of 20 trials, and face decoding was above chance level on 11 out of 20 trials. When looking at the volume-by-volume results, 36 out of 40 trials in total reached above chance level performance (i.e. above 0.5) at some time during the 24 s trial. The 4 trials that never reached above chance level decoding accuracy were all trials in which the object was associated with a face. A possible explanation could be that the memory performance for the face pairs was lower than the scene pairs at the group level (on average 85% correct for scenes, and 75% correct for faces).

Altogether, we conclude that it is feasible with the current set-up to use decoding accuracy as neurofeedback as a tool to train participants to associate stimuli.

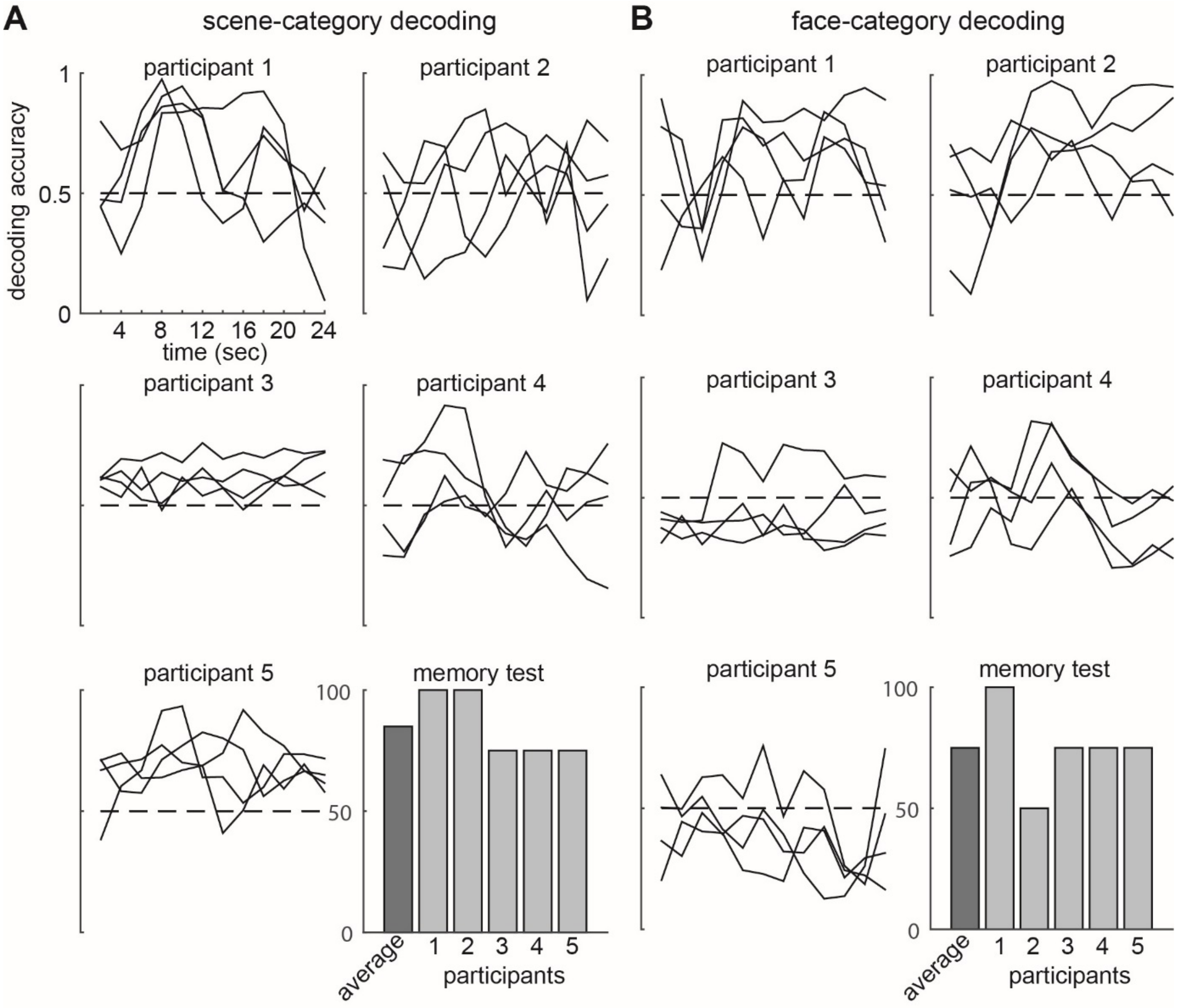

Supplementary Figure 2: Single subject, single trial, volume-by-volume scene-category (A) and face-category (B) decoding during the pilot experiment.

Each plot shows the decoding evidence (of scenes in A, and of faces in B) over time of a single participant for each trial separately (total of 4 trials per participant) during the pilot experiment (as described in supplementary material). During this decoding test phase, the participant was looking at objects that were associated with a scene (in A) or a face (in B) during a prior learning phase. At the bottom right, the memory performance for this category of the group on average (dark bar) as well as each participant separately (light bars) is displayed.

Methods

Participants

Seven students from the Radboud University campus in Nijmegen participated in this study. All participants were right-handed and had normal or corrected-to-normal vision. Two participants had to be excluded to do technical problems. Thus, five students (one male, aged 19-27 years, mean age 21.2) were included in this pilot study. All participants gave written informed consent. Participants were informed during the informed consent procedure that their data as acquired during the experiment could be used for publication or on conferences in an anonymized form. The study was approved by the local ethics committee (CMO, Arnhem/Nijmegen). All experiments were performed in accordance with the guidelines and regulations set by the local ethics committee (CMO, Arnhem/Nijmegen).

Task design
This pilot experiment consisted of four phases; a training phase, a learning phase, a test phase, and a memory test (see Supplementary Fig. 1). The training phase was used to train a classifier on brain activity patterns evoked by faces and scenes. The training phase consisted of 28 blocks in total, interleaved blocks with images of faces and blocks with images of scenes. Each block lasted for 30 seconds and was followed by a fixation cross which was presented for 12 seconds. Each block consisted of 14 unique pictures (i.e. each block had a different set of pictures), each picture was presented for 2 seconds, and, additionally, the first picture of the block was repeated at a random position within that block. Participants had to press a button when they saw the first picture being repeated. They were asked to maintain attending the images throughout the entire block. During the learning phase the participant was required to learn four object-face pairs, and four object-house pairs. Each pair was presented together on the screen 14 times (stimulus duration 1.5 sec, inter-trial interval 0.5 sec). During the test phase, participants were presented with the eight objects from the learning phase. Each object was presented in isolation, with the eight objects presented consecutively in a random order. Each object was presented only once for 24 sec. After the real-time fMRI session, participants were placed behind a computer screen, and conducted a memory test. During this (self-paced) memory test, participants were tested for their memory for the eight pairs. For each object, they were asked to which category this object belonged (face or scene), followed by a certainty rating (scale 1 to 4). Additionally, they were presented with all exemplars from the category used during the learning phase, and asked to identify the correct exemplar which was associated to this object, again followed by a certainty rating (scale 1 to 4).

Image acquisition and analyses
Image acquisition, real-time fMRI analysis, online image preprocessing, and online decoding was performed as described in the main methods section.
